# The effect of pre- and post-harvest sodium nitroprusside treatments on the physiological changes of cut *Alstroemeria aurea* ‘Orange Queen’ during vase life

**DOI:** 10.1186/s12870-024-05393-x

**Published:** 2024-07-17

**Authors:** Soheila Sadeghi, Zohreh Jabbarzadeh

**Affiliations:** Department of Horticultural Science, Faculty of Agriculture, Urmia University, Urmia, Iran

**Keywords:** Alstroemeria, Antioxidant enzymes, Cut flower, Ion leakage, Nitric oxide, Senescence, Vase life

## Abstract

Cut flowers deteriorate rapidly after harvest, lasting mere days. To extend their vase life, various postharvest techniques are employed. Due to limited knowledge about the postharvest physiology of Alstroemeria cut flowers and the specific role of secondary compounds and antioxidant systems in their protection, this study investigated the optimal dosage of sodium nitroprusside (SNP) as a nitric oxide (NO) donor to enhance quality and antioxidant defenses. Preharvest foliar application of SNP at 0, 50, 100, and 200 µM followed by short-term pulsing treatments upon harvest at the same concentrations were applied in a factorial design. Results revealed that a preharvest 100 µM SNP treatment combined with a 50 µM postharvest pulse significantly increased the total amount of phenols (over 20%), antioxidant capacity (more than doubled), and the activity of two antioxidant enzymes (ascorbate peroxidase by over 35% and guaiacol peroxidase by about 20%). Notably, this combination also diminished ion leakage (by about 20%), ultimately extending the vase life by more than 40% compared to untreated plants. Therefore, SNP application at these specific dosages proves effective in bolstering Alstroemeria cut flower quality and vase life through enhanced total phenols and a strengthened antioxidant system.

## Introduction

Alstroemerias, prized for their captivating beauty and diverse blooms, hold a significant presence in the global cut flower market [1]. Among ornamental plants, both as cut flowers and potted specimens, alstroemerias reign near the top. They boast a spectrum of vibrant colors, from sunshine yellows and soft pinks to fiery oranges, pristine whites, and captivating purples [2]. *Alstroemeria aurea*, a native of Chile and Argentina, exemplifies this splendor, gracing with its hardy yellow or yellowish-orange petals adorned with charming brown speckles [3].

The primary challenge in the cut flower industry, specifically in the postharvest phase, is the short vase life of flowers. This issue, along with early leaf yellowing and perianth abscission, significantly diminishes the economic value of flowers due to their accelerated senescence [4].

Various factors contribute to the short vase life of cut alstroemerias, including genotype, environmental conditions, oxidative stress, microorganisms, and sensitivity to ethylene. Oxidative stress, in particular, is a major factor that can lead to premature senescence and reduced vase life [4]. Phenolic compounds are secondary metabolites that have antioxidant properties. They can help to protect plants from oxidative stress and extend vase life [5].

Nitric oxide (NO), a crucial signaling molecule in both plants and animals, emerges as another promising ally. In plants, NO plays a vital role in various developmental stages and physiological processes, from orchestrating pollination and pollen tube growth to influencing seed germination, root development, and even regulating stomatal opening [6]. Beyond its inherent benefits, NO boasts environmental friendliness, making it an attractive tool for extending the postharvest life of diverse horticultural crops. Its protective abilities extend beyond senescence control, influencing processes like photosynthesis, pigment synthesis, and defense systems [7]. Notably, NO’s antagonistic relationship with ethylene makes it even more valuable. By reducing ethylene synthesis and activity, NO effectively combats premature aging in plants, evident in the delayed yellowing of leaves. Sodium nitroprusside (SNP), a widely used NO-releasing compound, has demonstrated its effectiveness in promoting longevity in cut flowers of various species [4].

Several studies have explored SNP’s potential in ornamental plants, highlighting its positive impact on quality and vase life. The potential benefits of SNP treatment include: Increased flower production, improved flower quality and prolonged vase life [9]. Previous research on gerbera cultivars revealed NO’s influence on multiple aspects, including extending vase life, enhancing the activity of antioxidant enzymes, and reducing stem neck issues [8]. Studies further demonstrate how SNP promotes the synthesis of beneficial compounds like total phenols and flavonoids, while simultaneously suppressing the activity of enzymes that degrade them [7]. This fortified antioxidant arsenal effectively combats the damaging effects of oxidative stress, leading to improved postharvest performance. Additionally, SNP treatment at optimal concentrations has been shown to maintain protein content in various cut flowers like rose (*Rosa hybrida* L.), lisianthus (*Eustoma grandiflorum*) and sunflower (*Helianthus annuus* L.), contributing to their extended longevity [4].

Limited research exists on how sodium nitroprusside (SNP) extends the vase life of cut flowers, particularly Alstroemeria. Exploring SNP’s effects represents a crucial area for the floriculture industry, potentially extending the vase life and beauty of Alstroemeria blooms after harvest. SNP’s ability to influence plant responses and delay senescence addresses a key need for sustainable and cost-effective treatments. This research delves deeper, comparing the effectiveness of various SNP concentrations and application methods (foliar application before harvest and pulse treatment after harvest) on Alstroemeria’s physiological and biochemical parameters. It specifically focuses on understanding how these chemicals influence petal senescence. The findings aim to significantly contribute to developing more effective SNP treatments. By understanding SNP’s influence on senescence, we may be able to extend the ornamental value and marketability of Alstroemeria flowers. This research holds significant promise for both advancing our scientific understanding of plant senescence and developing practical applications for the floriculture industry. In essence, this investigation focuses on mitigating postharvest senescence in *Alstroemeria aurea* cut flowers using SNP. The goal is to optimize the SNP dosage for efficient marketing through the development of compatible postharvest treatments.

## Materials and methods

### Plant materials and growing conditions

- **Plants**: Alstroemeria plants (*Alstroemeria aurea*) cv. Orange Queen (Royal Van Zan Ten Netherlands) were obtained from a commercial greenhouse.
- **Growing conditions**: Plants were grown in plastic pots with a diameter of 24 cm and a height of 19 cm. The soilless growth medium consisted of perlite: cocopeat (1:3 v/v). The day/night temperature of the greenhouse was set at 18–21/10–12 °C, 10–12 h of light duration, and 400–500 µmol/m^2^/s light intensity.

### Treatment with SNP

**Fig. 1:**
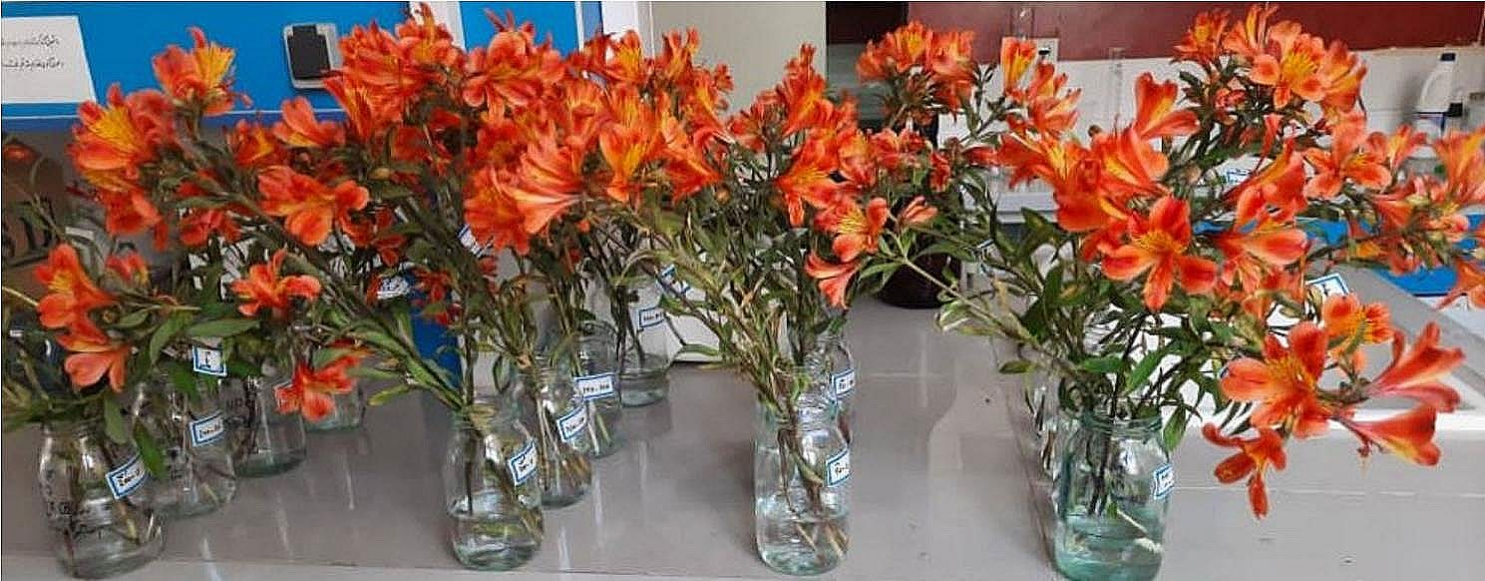
Alstroemeria cut flowers treated with SNP

- **Pre-harvest application**: Pre-harvest foliar spraying was done at two-week intervals for four months (a total of 8 times), starting about one month after the plants were established in the greenhouse (about 20 cm height). The plants were sprayed with SNP (Fluka company-Switzerland) at concentrations of 0, 50, 100, or 200 µM.
- **Post-harvest application**: Two weeks after the last spray, flowers were harvested at the bud stage, when the main florets displayed initial color and were nearing bloom. For the postharvest treatment, the flowers underwent a 24-hour pulse treatment in the lab after harvest. The cut flowers were harvested early in the morning and immediately placed in buckets containing tap water and transported directly to the laboratory. The flower stems were re-cut under water to a 40-cm length. The SNP solution was prepared by dissolving sodium nitroprusside powder (Fluka company-Switzerland) in distilled water at concentrations of 50, 100, or 200 µM. The solution was used immediately due to its short half-life. Four flower stems were placed in each 500 ml flask containing the treatment solution for 24 h, allowing for uptake by the stems. Afterward, the stems were transferred to vases filled with a solution of distilled water and 4% sucrose, where they remained until the end of their vase life.
- **Vase life conditions**: Throughout the experiment, the flowers were kept under controlled conditions: a constant temperature of 22 °C ± 1 °C, relative humidity around 70%, and a 12-hour light cycle provided by fluorescent lamps with a light intensity of 13 µmol/m²/s (Fig. 1).

### Electrolyte leakage

Membrane stability of the petals was determined by measuring their electrolyte leakage. Petal disks (0.2 g) were washed in distilled water and incubated in 15 ml of distilled water at 40 °C for 30 min. The initial conductivity (EC1) of the solution was measured. Subsequently, the petal disks were boiled with the solution at 100 °C for 10 min, cooled to room temperature, and the final conductivity (EC2) was measured again. Electrolyte leakage (%) was calculated using the following formula [10]:

EL= (EC1/EC2) ×100

### Antioxidant capacity

Antioxidant activity was measured as the inhibition of 1,1-diphenyl-2-picrylhydrazyl hydrate (DPPH) radicals according to Nakajima et al. [11]. Freshly prepared petal extract (100 µl) was mixed with 1900 µl DPPH and kept in the dark at room temperature for 30 min. Absorbance was then measured at 517 nm. Inhibition percentage, an indicator of antioxidant capacity, was calculated as:

DPPH-scavenging effect (%) = Ac-As/Ac*100

Ac = Control absorption

As = Sample absorption

### Total phenol content

Total phenol content was determined using the method of Marinova et al. [12] with sodium carbonate and Folin-Ciocalteu reagent. Petal tissue was homogenized in 85% methanol, centrifuged at 5000 × g for 5 min at 4 °C, and incubated at 20 °C for 30 min. Absorbance was then measured at 750 nm. Total phenol concentration was expressed as milligrams of gallic acid equivalents per gram of fresh petals.

### Plant extract preparation for enzyme activity measurement

The methods of Kang and Saltivite [13] were used to prepare plant extracts for determining the activity of ascorbate peroxidase and guaiacol peroxidase enzymes. Fresh petal tissue (0.5 g) was homogenized in 3 ml of Tris buffer (pH 7.5) containing 0.5 M hydrochloric acid, 3 mM magnesium chloride, and 1 mM EDTA. For ascorbate peroxidase activity measurement, the extraction buffer also contained 0.2 mM ascorbate. The homogenate was centrifuged at 4000 rpm for 4 min at 4 °C. The resulting supernatant was used as the crude extract for measuring antioxidant enzyme activity.

#### Guaiacol peroxidase (GPX) activity

The reaction mixture contained 1 ml of 1% guaiacol, 1 ml of 1% hydrogen peroxide, 2.5 ml of 50 mM phosphate buffer (pH 5.7), and 0.1 ml of the crude extract. Guaiacol peroxidase activity was determined by monitoring the increase in absorbance at 420 nm (A420) for 1 min using a spectrophotometer. The extinction coefficient of 26.6 cm^-1^ mM^-1^ was used for activity calculations [14].

$$\:unit\:\frac{mM}{min}=\:\frac{do\:D/\text{min}\left(slope\right)\times\:vol.\:of\:assay\:}{Extinction\:coefficient\:\left(26.6\right)}$$

#### Ascorbate peroxidase (APX) activity

Ascorbate peroxidase activity was measured using the method of Nakano and Asada [15]. The reaction solution contained 2.5 ml of 50 mM phosphate buffer (pH 7) with 0.1 mM EDTA, 1 mM sodium ascorbate, 0.2 ml (200 µl) of 1% H_2_O_2_, and 0.1 ml (100 µl) of the crude extract. Ascorbate peroxidase activity was assessed by measuring the decrease in absorbance at 290 nm (A290) during one minute using a spectrophotometer. The extinction coefficient of 2.8 mM^-1^ cm^-1^ was used for activity calculations.

$$\:unit\:\frac{mM}{min}=\:\frac{do\:D/\text{min}\left(slope\right)\times\:vol.\:of\:assay\:}{Extinction\:coefficient\:\left(2.8\right)}$$

### Vase life

Vase life, a crucial factor for marketability, refers to the duration cut flowers retain their commercial and aesthetic appeal. In this study, Alstroemeria vase life ended when 50% of the leaves yellowed or 50% of the flowers wilted and fell, aligning with established criteria [16, 17]. To monitor this, flower health was assessed daily based on these same visual cues.

### Statistical analysis of data and software used

This experiment was conducted using a factorial design with three factors including pre-harvest foliar application of SNP with 0, 50, 100 or 200 µM as a first factor, post-harvest pulsing treatment of SNP with concentrations of 0, 50, 100 or 200 µM as a second factor and times of evaluation during vase life period (first, 5th and 12th day) as a third factor. This experiment was done with four replicates per treatment. Data were managed and analyzed using SAS version 9.2. Means were compared using Tukey’s multiple range test at the 1% probability level.

## Results

### Total phenol

**Fig. 2:**
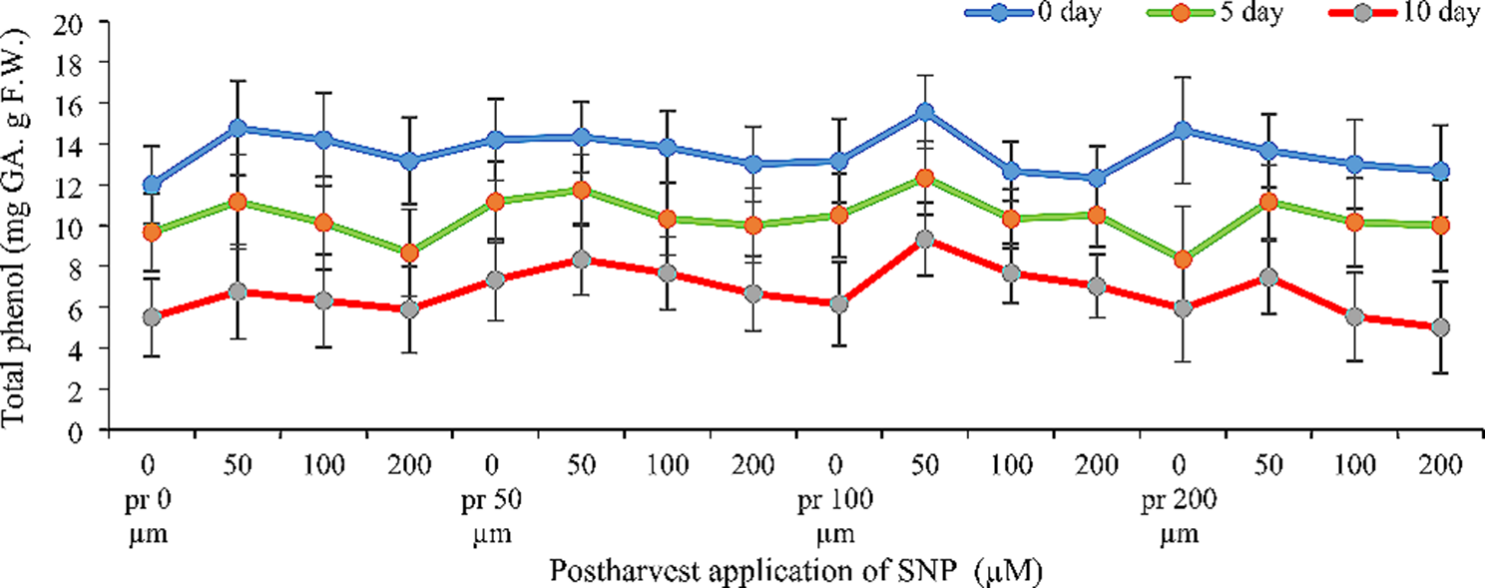
Effect of pre- and post-harvest application of SNP during vase life period on total phenol content of Alstroemeria flower ‘Orange Queen’ (pr: preharvest application of SNP)

The application of SNP, both pre- and post-harvest, increased total phenol content in Alstroemeria cut flowers. However, the amount of phenol decreased over time for all treatments. Interestingly, the total phenol content in petals did not differ significantly between pre-harvest and post-harvest SNP application (Fig. 2). However, the various SNP concentrations had varied effects. Concentrations of 50 and 100 µM SNP helped maintain total phenol content throughout the observation period (three sampling times) compared to the control group. Specifically, compared to the control, 100 µM SNP pre-harvest treatment and 50 µM SNP post-harvest treatment increased total phenols by about 30%, 28%, and 70% on the first, fifth, and tenth days, respectively (Fig. 2).

### Antioxidant capacity

**Fig. 3:**
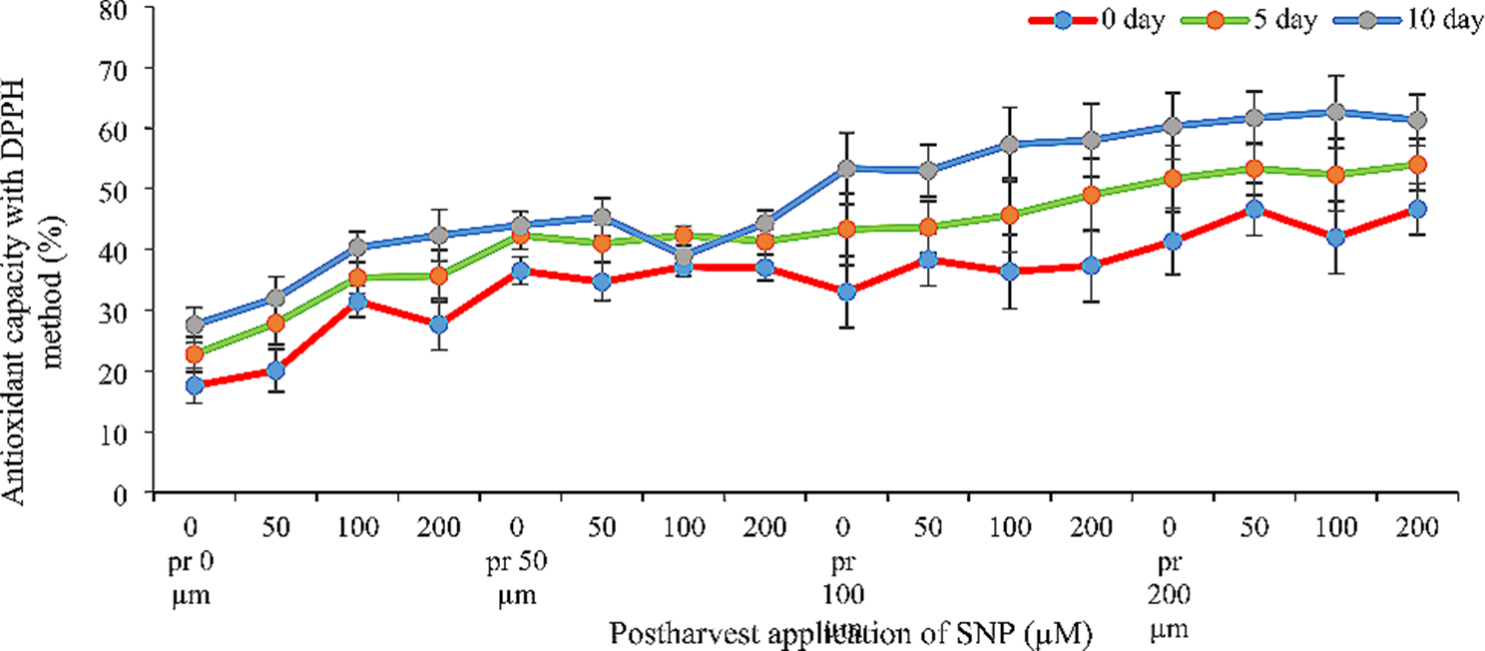
Effect of pre- and post-harvest application of SNP during vase life period on the antioxidant capacity (DPPH) of Alstroemeria flower ‘Orange Queen’ (pr: preharvest application of SNP)

All SNP concentrations significantly boosted the antioxidant capacity of Alstroemeria cut flowers. Notably, a synergistic effect was observed when SNP was applied both before and after harvest, leading to greater increases compared to single applications. Higher SNP concentrations were generally more effective. Pre-harvest application of 100 and 200 µM SNP showed the most significant improvement. Interestingly, all three concentrations (50, 100, and 200 µM) were effective in enhancing antioxidant capacity when applied after harvest. For instance, the combination of 200 µM SNP pre-harvest and 100 µM SNP post-harvest more than doubled the antioxidant capacity at all three measured time points (days 0, 5, and 10) compared to the control group (Fig. 3).

### Guaiacol peroxidase activity (GPX)

**Fig. 4:**
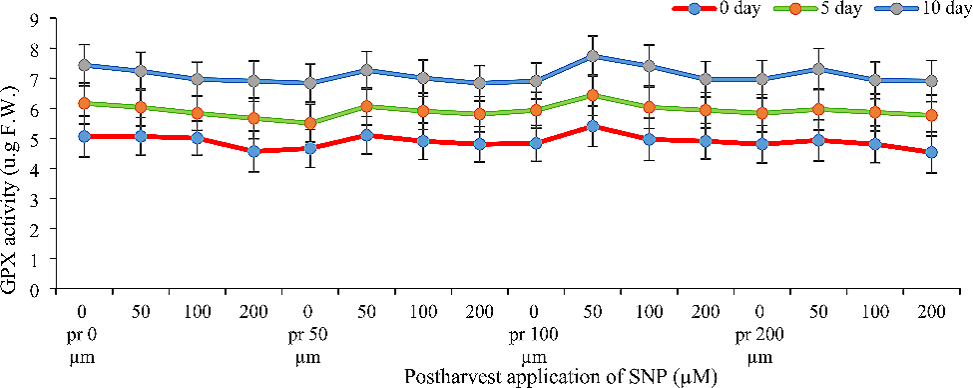
- Effect of pre- and post-harvest application of SNP during vase life period on the guaiacol peroxidase activity of Alstroemeria flower ‘Orange Queen’ (pr: preharvest application of SNP)

Similar to the observed antioxidant capacity, SNP application synergistically increased GPX activity, regardless of pre-harvest or post-harvest application. However, the concentration of SNP mattered. While application timing didn’t significantly affect GPX activity, different SNP concentrations had varying effects (Fig. 4). The most significant increase in GPX activity (25%, 18%, and 19% at days 0, 5, and 10, respectively) was achieved with a combined treatment: 100 µM SNP pre-harvest and 50 µM SNP post-harvest (Fig. 4).

### Ascorbate peroxidase activity (APX)

**Fig. 5:**
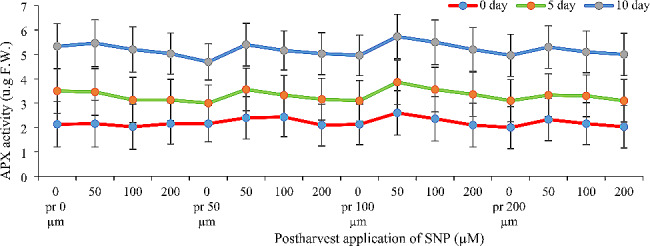
- Effect of pre- and post-harvest application of SNP during vase life period on the ascorbate peroxidase activity of Alstroemeria flower ‘Orange Queen’ (pr: preharvest application of SNP)

SNP application influenced APX activity, but pre-harvest vs. post-harvest application didn’t show a significant difference. Interestingly, APX activity increased naturally over time. Additionally, SNP concentration played a role (Fig. 5). The most pronounced increase in APX activity was observed with the same combined treatment that benefited GPX activity: 100 µM SNP pre-harvest and 50 µM SNP post-harvest. This treatment resulted in significant increases of 53%, 35%, and 45% on days 0, 5, and 10, respectively (Fig. 5).

### Electrolyte leakage

**Fig. 6:**
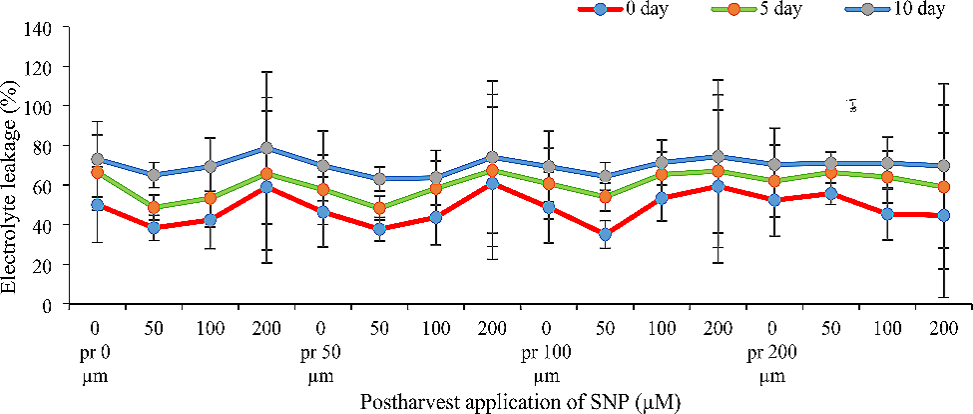
Effect of pre-and post-harvest application of SNP during vase life period on electrolyte leakage rate of Alstroemeria ‘Orange Queen’ (pr: preharvest application of SNP)

As expected, ion leakage increased over time, with the highest levels observed on day 10 (Fig. 6). SNP application, both pre-harvest and post-harvest, effectively prevented this rise in ion leakage. Interestingly, the timing of SNP application (pre-harvest vs. post-harvest) didn’t significantly impact the results. In both cases, SNP treatment reduced ion leakage. However, the concentration of SNP was crucial. Notably, 200 µM SNP was counterproductive, actually increasing ion leakage to similar levels as the control group without SNP. The most effective treatment for reducing ion leakage was the combination of 100 µM SNP pre-harvest and 50 µM SNP post-harvest. This combination significantly reduced ion leakage by 1.43, 1.23, and 1.14 times on days 0, 5, and 10, respectively, compared to the control.

**Fig. 7:**
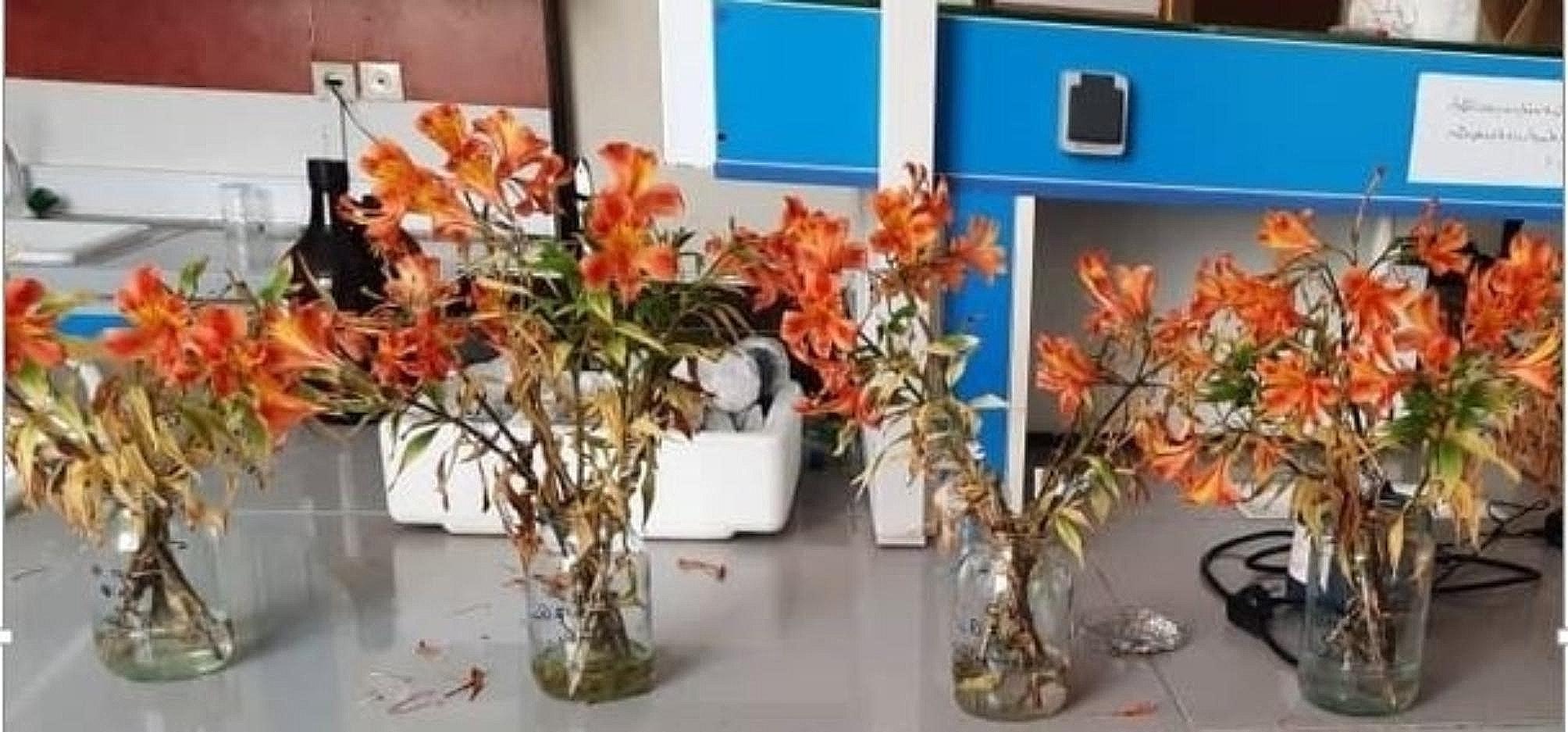
Effect of pre and postharvest application of SNP on alstroemeria’s cut flowers. (from left to right; control plant, SNP 100 µM pre + 50 µM post, SNP 100 µM pre + 100 µM post, SNP 100 µM pre + 200 µM post). (pre: preharvest; post: postharvest)

Generally, in this experiment, treatment with 50 µM SNP post-harvest and 100 µM SNP pre-harvest had the best effects on physicochemical properties of alstroemeria cut flowers (Fig. 7).

### Vase life

**Fig. 8:**
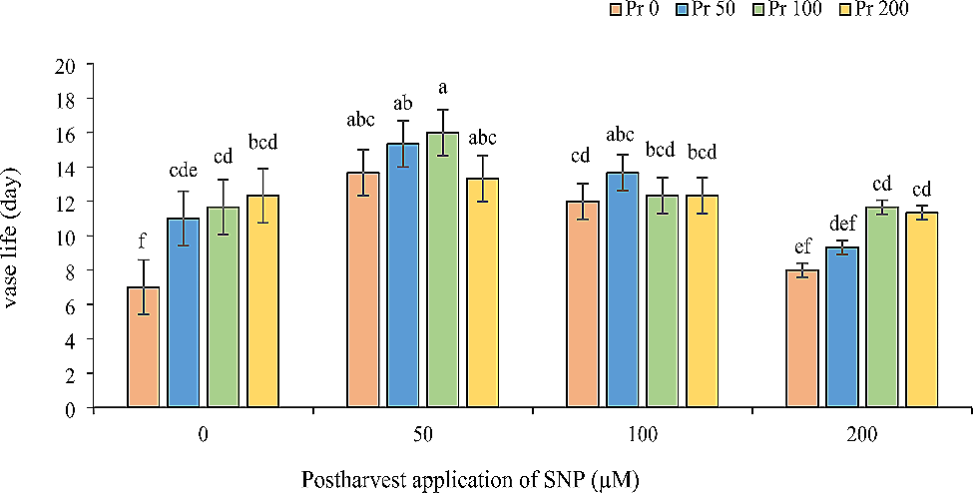
Effect of pre- and post-harvest application with different concentrations of SNP on vase life of Alstroemeria ‘Orange Queen’ (pr: preharvest application of SNP (µM). Different letters indicate statistically significant differences between the treatments and control in (*P* ≤ 0.01)

Sodium nitroprusside (SNP) application significantly extended the vase life of Alstroemeria cut flowers. Interestingly, the timing of application (pre-harvest vs. post-harvest) did not significantly influence the results. All three SNP concentrations tested were effective in increasing vase life. The most successful treatment involved a combination of 100 µM SNP pre-harvest and 50 µM SNP post-harvest, achieving a vase life of 16 days. This represents a remarkable 44% increase compared to the control group (Fig. 8).

## Discussion

### Total phenol

Figure 2 shows that pre- and post-harvest application of SNP significantly increased phenolic content in Alstroemeria petals. Phenolics, a diverse group of plant compounds including flavonoids and anthocyanins, offer broad benefits in food, medicine, and other industries [18]. Their antioxidant and anti-inflammatory properties are particularly valuable. Phenols combat free radicals through various mechanisms, like scavenging them, converting primary oxidation products, and chelating metals [19, 20]. However, an enzyme called polyphenol oxidase can degrade phenols, reducing their antioxidant power and shortening the shelf life of fruits and flowers [21]. Interestingly, our study suggests that SNP might increase phenol content by boosting phenylalanine ammonia-lyase (PAL), the key enzyme in their biosynthesis, while possibly suppressing polyphenol oxidase activity. This aligns with research by Kazemzadeh Beneh et al. [22] in gladiolus, Shabanian et al. [7] in gerbera and Ul-Haq in Consolida [23] who found that SNP increased phenols and reduced polyphenol oxidase activity in cut flowers and lengthening their vase life. Notably, higher phenolic content generally correlates with longer vase life.

### Antioxidant enzymes activity and antioxidant capacity

Figures 3, 4 and 5 demonstrate that applying sodium nitroprusside (SNP) pre- and post-harvest significantly boosted both antioxidant capacity and enzyme activity in Alstroemeria petals. In cut flowers, reactive oxygen species (ROS) production is a major culprit behind damage, altering membrane compounds and antioxidants [24]. Antioxidants delay oxidation by either initially inhibiting or preventing excessive free radical formation during reactions [25]. ROS accumulation in plants triggers a secondary stress called oxidative stress, which plants combat through various mechanisms including their inherent antioxidant system [26]. This system comprises two pathways: enzymatic (e.g., superoxide dismutase, catalase, ascorbate peroxidase) and non-enzymatic antioxidant defenses within plant cells [27]. Nitric oxide acts as both an antioxidant and inducer of antioxidant enzymes, effectively reducing free radicals and delaying senescence [28]. This explains our findings – SNP likely boosted antioxidant capacity and enzyme activity by both directly scavenging ROS and enhancing the plant’s own defenses. The use of nitric oxide varies depending on the species, variety and concentration used, but it has been shown that nitric oxide in low concentrations leads to the expression of genes involved in the synthesis of protective enzymes [29]. The increase of antioxidant enzymes such as ascorbate peroxidase and guaiacol peroxidase as well as the increase of antioxidant capacity in the present study may be due to the above reasons. Various studies have shown that nitric oxide after reacting with reactive oxygen species leads to the production of peroxynitrite and reducing the production of this ROS. When the pH is within the physiological range, peroxynitrite production can dissociate into a nitrate anion and a proton, or it can react with a hydrogen peroxide radical, thereby reducing ROS [29], which is probably the reason for the increase in antioxidant capacity and activity of antioxidant enzymes in the present study. In many similar studies, an increase in the activity of antioxidant enzymes has been reported. For example, Naing et al. [30] reported the positive effect of nitric oxide on the activity of antioxidant enzymes on gerbera cut flowers. In another study, Mittal and Shalini [31] reported that the application of sodium nitroprusside on gladiolus flowers increases the activity of antioxidant enzymes such as catalase and ascorbate peroxidase, which is consistent with the results of the present study. In the present study, it was shown that the use of high concentrations (200 µM) of sodium nitroprusside (both in pre- and post-harvest stages) decreases the activity of antioxidant enzymes and antioxidant capacity; nitric oxide in high concentrations appears to release cyanide and toxicity in plants through reaction with superoxide anion [28]. High concentrations of nitric oxide have now emerged, with oxidative stress, as a major arbiter of plant programmed cell death (PCD); however, both cytotoxic and cytoprotecting/stimulating properties of NO have been described in plants. High levels of NO are associated with cell death and DNA fragmentation in Taxus cultures [32]. An increase in NO levels has also been associated with the progression of natural senescence and cytokinin-induced senescence [33], suggesting its involvement in the modulation of these physiological processes as well.

### Ion leakage

The study further revealed that SNP reduced ion leakage rate, with the decrease trending upwards throughout the vase life (Fig. 6). Ion leakage refers to cell wall breakdown or the discharge and leakage of cell contents into the extracellular environment. As senescence progresses, electrolyte leakage increases, alongside the activity of antioxidant enzymes like lipoxygenase. This leads to cell membrane depletion and ion leakage, ultimately shortening the flower’s vase life. Nitric oxide-releasing compounds like SNP increase membrane permeability, maintain solution pH [27], directly interact with lipid peroxyl radicals, and indirectly inhibit lipoxygenase by reducing Fe^3+^ to Fe^2+^ in its active site [4]. This disrupts reactive oxygen species, maintaining membrane stability and reducing ion leakage [27]. The rapid reaction of SNP with alkoxy lipids and peroxide radicals is another mechanism for reducing ROS, preventing free radical release and lipid oxidation [28, 34]. Many studies have shown that SNP reduces ion leakage, leading to decreased transpiration and increased stomata closure, ultimately extending the flower’s vase life [35]. Our study confirms this, showing that pre- and post-harvest SNP application reduced ion leakage. Similarly, Abbasi et al. [36] reported increased membrane stability and decreased ion leakage in cut gerbera flowers treated with 100 µM SNP. Mousavi et al. [37] also observed reduced ion leakage in *Echinacea angustifolia* under drought stress conditions thanks to SNP application. Additionally, Mirzaei Esgandian and Jabbarzadeh [38] reported reduced ion leakage in two rose cultivars, Utopia and Dolce Vita, due to SNP application. Nitric oxide’s dual function as a strong oxidant or effective antioxidant primarily depends on its concentration, environmental conditions, tissue and cell conditions, and other hormone concentrations. Applying high SNP concentrations disrupts normal plant metabolism and damages membranes, proteins, and nucleic acids [39]. This likely explains the observed increase in ion leakage at high concentrations in our study.

### Vase life

This study demonstrates that sodium nitroprusside (SNP) treatment successfully extended the vase life of Alstroemeria cut flowers. A major challenge in post-harvest storage of cut flowers is senescence, the aging process. During senescence, the formation of reactive oxygen species (ROS) causes oxidative damage, indicating the weakening of the plant’s natural antioxidant defenses [40]. Aging in cut flower petals is also associated with water imbalance and water loss, leading to wilting and reduced marketability [41]. Our research found that applying 100 µM SNP pre-harvest and 50 µM SNP post-harvest decreased water loss and ion leakage in Alstroemeria, while simultaneously extending vase life. This suggests that maintaining water balance is crucial for extending vase life and marketability. Previous research suggests nitric oxide (released by SNP) delays senescence by scavenging ROS, reducing membrane damage, and promoting the production of antioxidant enzymes [42]. Our findings support this, as SNP application increased antioxidant enzyme activity, antioxidant capacity, and ultimately, vase life in Alstroemeria. These results align with studies on gladiolus by Dwivedi et al. [27] and Kazemzadeh-Beneh et al. [22].

## Conclusion

This study explored the use of sodium nitroprusside (SNP) to improve the quality of Alstroemeria cut flowers after harvest. Our findings revealed that SNP application, both before and after harvest, significantly increased the total amount of phenols and reduced ion leakage from the cells. These results suggest enhanced cell stability and reduced free radical activity, key indicators of delayed senescence. The data further supports the notion that SNP bolsters the flower’s antioxidant system. We observed a significant increase in both overall antioxidant capacity and the activity of specific antioxidant enzymes within the Alstroemeria flowers treated with SNP. Notably, the most effective combination involved applying 100 µM SNP pre-harvest followed by 50 µM SNP post-harvest. These findings strongly suggest that SNP has promising potential as a novel treatment to extend the vase life of cut flowers. Further research is necessary to refine the optimal concentration and application methods of SNP for achieving maximum effectiveness.

## Data Availability

The datasets used in this paper are available from the first author on reasonable request.

## Abbreviations

SNP: Sodium nitroprusside
NO: Nitric Oxide
EL: Electrolyte Leakage
DPPH: 1,1-diphenyl-2-picrylhydrazyl hydrate
GPX: Guaiacol peroxidase
APX: Ascorbate peroxidase
